# Factors associated with food insecurity in children and adolescents with type 1 diabetes mellitus

**DOI:** 10.1016/j.jped.2026.101589

**Published:** 2026-07-24

**Authors:** Gabriel França Toledo Pinto, Juliana Silva do Nascimento Braga, Ana Beatriz Guterres Azevêdo Mathias, Pétala Machado Sizisnande, Pedro Henrique Vidal Rodrigues, Jorge Luescher, Patricia de Carvalho Padilha

**Affiliations:** a Universidade Federal do Rio de Janeiro (UFRJ), Instituto de Nutrição Josué de Castro (INJC), Instituto de Puericultura e Pediatria Martagão Gesteira (IPPMG), Programa de Pós-Graduação em Saúde Materno Infantil, Núcleo de Estudos em Nutrição e Pediatria (NUTPED), Rio de Janeiro, RJ, Brazil; b Universidade Federal do Rio de Janeiro (UFRJ), Instituto de Nutrição Josué de Castro (INJC), Núcleo de Estudos em Nutrição e Pediatria (NUTPED), Rio de Janeiro, RJ, Brazil; c Instituto de Puericultura e Pediatria Martagão Gesteira (IPPMG), Universidade Federal do Rio de Janeiro(UFRJ), Rio de Janeiro, RJ, Brazil

**Keywords:** Food insecurity, Child nutrition, Type 1 diabetes mellitus, Sociodemographic factors

## Abstract

**Objective:**

To evaluate the factors linked to Food Insecurity (FI) in children and adolescents with Type 1 Diabetes Mellitus (DM1).

**Methods:**

A cross-sectional study was conducted with children and adolescents at the Diabetes Outpatient Clinic of the IPPMG/UFRJ. Procedures included assessing FI levels using the Brazilian Food Insecurity Scale (EBIA). Sociodemographic data collected included age, gender, number of family members, caregiver's education, social benefits, and income. A 5% significance level was used, with 95% confidence intervals.

**Results:**

The sample included 130 children and adolescents, mostly female (60.8%, n = 79), and most did not receive social benefits (63.8%, n = 81). In most cases, the mother was the primary caregiver (77.7%, n = 101), and the caregivers had at least completed high school (80%, n = 104). Most participants were eutrophic based on BMI/age and sex (58.4%, n = 76) and had appropriate height for their age (96.9%, n = 126). The prevalence of FI was 67.4% (n = 87), with 50.4% (n = 65) experiencing mild FI, 11.6% (n = 15) moderate FI, and 5.4% (n = 7) severe FI.

**Conclusion:**

A significant association was found between FI and income, caregiver's education, and receipt of benefits. The study highlights income as a key factor in food acquisition. These findings stress the need for intersectoral actions to develop public policies and programs aimed at reducing poverty, promoting food security, and ensuring adequate, healthy nutrition, especially within this population.

## Introduction

Diabetes mellitus (DM) is a group of chronic diseases in which affected individuals mainly experience hyperglycemia. This increase in blood glucose levels can result from insufficient insulin secretion or resistance to the hormone's action [1] If not properly managed, this condition can lead to cardiovascular complications, neuropathies, nephropathies, and other issues [2]

The most recognized types of Diabetes are: Type 1 Diabetes (DM1), Type 2 Diabetes (DM2), and Gestational Diabetes (GDM). In Type 1 Diabetes, hyperglycemia occurs due to inadequate insulin production caused by the destruction of pancreatic beta cells, often through an autoimmune process [1,3]

According to the American Diabetes Association[1], individuals with Diabetes need to maintain a healthy diet, consisting of a variety of nutrient-rich foods and limited in added sugars and fats. This approach can help control Glycated Hemoglobin (HbA1c), cholesterol, and blood pressure, while also preventing or delaying comorbidities and complications related to Diabetes. However, factors such as low income and difficulty accessing healthy foods can hinder these individuals' ability to follow a healthy diet [4]

The concept of food security, as defined by the Organic Law on Food and Nutrition Security, is “the right of everyone to regular and permanent access to quality food, in sufficient quantity, without compromising access to other essential needs, based on health-promoting eating practices that respect cultural diversity and are socially, economically, and environmentally sustainable." When this right is not guaranteed, food insecurity occurs, characterized by a lack of access to quality food in adequate amounts, mainly due to income and availability issues [5]

The Food and Agriculture Organization (FAO) estimates that approximately 733 million people experienced hunger in 2023, a 152 million increase compared to 2019 [6] Regarding children under 5 years old, 1 in 4 children worldwide live in severe child poverty, totaling 181 million children in this age group. These children typically consume only two food groups daily. In Brazil, 8% of children in this age range live under similar conditions [7]

The 2022 FAO global report on the State of Food Security and Nutrition estimates that between 2019 and 2021, Brazil had a prevalence of 7.7% (approximately 15.4 million people) experiencing severe food insecurity, and 18.3%. Approximately 37.5 million people (3%) experience moderate food insecurity [8]

This scenario is worrying, considering that food insecurity can represent several harms to the lives of individuals, with its most serious consequence being hunger. This lack of food is even more harmful in more vulnerable groups, such as children and adolescents. In addition, people with chronic diseases in a situation of Food Insecurity tend to have a worsening of their health conditions [9,10]

For individuals with diabetes, food insecurity is related to poorer control of the disease through 3 main pathways: nutritional, behavioral, and mental health, as discussed by Weiser et al. (2015) [11] Factors such as low income, high food prices, lack of time to buy and prepare food, and limited access to establishments that sell healthy foods are described as obstacles to consuming a nutritious diet, which can lead to food insecurity [12]

As seen, this topic is of great relevance for the proper management of the disease. Despite this, there is a shortage of studies addressing the factors associated with food insecurity in children and adolescents with type 1 diabetes mellitus. Adequate food in terms of quantity and quality is essential for the proper management of the disease and satisfactory development of this population. Added to this is the fact that food insecurity is a violation of a right.

Due to this scenario, the objective of this study was to evaluate the factors associated with food insecurity in children and adolescents with type 1 diabetes treated at the Diabetes Outpatient Clinic of the Instituto de Puericultura e Pediatria Martagão Gesteira (IPPMG) at the Federal University of Rio de Janeiro.

Considering the socioeconomic challenges faced by many families of children and adolescents with type 1 diabetes mellitus, the authors hypothesized that food insecurity would be highly prevalent in this population and would be associated with lower household income, lower caregiver educational level, and receipt of social benefits.

## Methods

### Study design

This is a cross-sectional study conducted at the Diabetes Mellitus Outpatient Clinic of IPPMG, at the Federal University of Rio de Janeiro. As the largest pediatric type 1 diabetes mellitus (T1DM) referral center in the city of Rio de Janeiro, the clinic receives patients from diverse socioeconomic backgrounds and multiple municipalities across the metropolitan region. Patients are treated by a multidisciplinary team through integrated consultations conducted by doctors, nutritionists, social workers, psychologists, and nurses. At the time of data collection, the sector had exactly 520 registered patients.

### Populations, sample and eligibility criteria

The sample size calculation considered an expected prevalence of food insecurity of 27.5%, based on previous literature, and margins of error of 10%, defining the minimum sample size as 49 patients [13] A sample size calculation was performed to ensure sufficient statistical power for detecting associations between food insecurity and sociodemographic factors. The final sample comprised 130 participants, substantially exceeding the minimum required sample size. Considering the difference between the prevalence observed in the study sample and the national reference prevalence, the effect size calculated using Cohen’s h was 0.82, indicating a large effect. A post hoc power analysis demonstrated statistical power greater than 99% to detect this difference, assuming a significance level of 5%.

Participants were recruited consecutively during routine outpatient visits, and all eligible patients attending the clinic during the data collection period were invited to participate.

The eligibility criteria were: (1) age between 2 and 16 years; (2) having been diagnosed with DM1 for at least 1 year; (3) absence of other autoimmune diseases, such as celiac disease. Children and adolescents using medications that affect weight gain, such as atypical antipsychotics and corticosteroids, and those with other diseases that require dietary restrictions were excluded. Data collection for the study was conducted using a specific research form, which was completed by researchers affiliated with the Center for Studies in Nutrition and Pediatrics (NUTPED) and had received proper training.

### Dependent variable (outcome)

Food insecurity - The tool used to assess the level of Food Insecurity (FI) was the Brazilian Food Insecurity Scale (EBIA)[14], which is a questionnaire consisting of 14 questions that can be answered with “yes” or “no”, of which six questions refer to family members under 18 years of age. The questions address issues such as concerns about food running out before it is possible to buy more, and the total absence of food. All of this refers to the three months before the interview. For each positive answer, a value of 1 (one) was assigned, and for each negative answer, a value of 0 (zero). The interviewee who answered negatively to all the questions was classified as having food security. Those who answered positively to up to 5 (five) questions were classified as having mild food insecurity. Between 6 (six) and 9 (nine) positive answers, the classification was moderate food insecurity. And from 10 (eleven) to 14 (fifteen) positive answers, severe food insecurity.

For statistical analyses, food insecurity categories were grouped into mild food insecurity and moderate/severe food insecurity.

### Independent variables

Sociodemographic and outpatient follow-up data

The sociodemographic data collected included: age of the child or adolescent (in years and months), sex (female or male), number of people in the family, education level of the main caregiver, income, and receipt of social benefits. Income was assessed by consulting the guardian and analyzed in terms of minimum wages, justifying the investigation based on its relationship with levels of food insecurity. The information collected regarding outpatient follow-up included: time since diabetes diagnosis (in years and months), age at diagnosis (in years and months), insulin regimen and dose per kilogram of ideal weight, nutritional status (as measured by BMI), and glycemic control.

To obtain the measurement of body mass, a FILIZOLA® PL 180 digital scale was used, with a maximum capacity of 150 kg and accuracy of 0.1 kg for children aged two and over and adolescents. Height (in meters) was measured using a TONELLI® wall-mounted stadiometer with an accuracy of 0.1 cm and performed in duplicate to reduce intra- and interpersonal variations, with the mean being calculated [15] The anthropometric indices height/age and body mass index (BMI)/age were assessed continuously, calculating the child's z-score, using as reference the growth standard proposed by the World Health Organization (WHO) for children aged 0 to 5 and 5 to 19 years [16]

For calculations, the WHO Anthro software (version 3.2.2, 2011, World Health Organization, Geneva, Switzerland) was used for children aged 0 to 5 years and the WHO Anthro Plus for children and adolescents aged 5 to 19 years (WHO 2009).

In categorical form, BMI-for-age, for children aged 0 to 5 years, was classified according to the cutoff points for severe thinness, below −3 z-score; thinness, between −3 and 2 z-score; eutrophy, between −2 and +1 z-score; risk of overweight, between +1 and +2 z-score; overweight, between +2 and +3 z-score; obesity, above +3 z-score. And for children and adolescents aged 5 to 19 years, they were classified according to the cutoff points for severe thinness, below −3 z-score; thinness, between −3 and 2 z-score; eutrophy, between −2 and +1 z-score; overweight, between +1 and +2 z-score; obesity, between +2 and +3 z-score; severe obesity, above +3 z-score [17]

Because overweight and obesity may influence dietary patterns, food acquisition, and metabolic control in individuals with T1DM, BMI-for-age classification was included as a potential factor associated with food insecurity. Overweight, obesity, and severe obesity categories were grouped as "excess weight" for analytical purposes.

Glycemic control was assessed according to the HbA1c value (%). This test was performed using the high-performance liquid chromatography (HPLC) method. The Endpoint test, VITROS System 5600, 4600, 5.1, FS, was used to determine HbA1c. Good glycemic control was considered when HbA1c was <7.5% [1]

### Statistical analysis

All statistical analyses were performed using the Statistical Package for the Social Sciences® software, version 26 for Windows (SPSS®, Inc., Chicago, IL). The Kolmogorov-Smirnov test was used to assess the distribution of variables. Continuous variables were shown to have a normal distribution and were therefore described as mean and standard deviation (SD). Categorical variables were described using absolute (n) and relative (%) frequencies.

The chi-square test was used to compare the proportions of categorical variables. The Student's *t*-test for independent samples was used to compare means. A significance level of 5% was adopted for all tests.

### Ethical aspects

The study complies with the ethical principles of non-maleficence, beneficence, justice and autonomy, contained in resolution 466/12 of the National Health Council and its complementary resolutions (CNS, 2012), and was approved by the Ethics Committee of the Instituto de Puericultura e Pediatria Martagão Gesteira (IPPMG) under opinion 6572367. At the beginning of data collection, after a brief explanation of the aspects involved in the research, the Term of Assent was read to children aged 7 and older and adolescents. The Term of Free and Informed Consent was requested from the guardians who, upon agreeing to participate in the research, signed it. For all the terms, two copies were requested, one from the researcher and one from the research participants.

## Results

The final sample analyzed consisted of 130 children and adolescents after applying the exclusion criteria. The mean age was 11.0 ± 3.6 years and months. The mean income found was 2.0 ± 0.8 minimum wages. As shown in Table 1, females (60.8%, n = 79) were predominant. The majority did not receive social benefits (63.8%, n = 81). In most cases, the mother was the main caregiver (77.7%, n = 101) and the guardian had at least started high school (80%, n = 104).

**Table 1:** Sociodemographic characteristics, glycemic control, nutritional status and food insecurity levels of children and adolescents aged 2 to 16 years with T1DM.

Variables	Total n		%
			
***Sex***			
*Female*	79		60.8%
*Male*	51		39.2%
			
***Benefit Receipt***			
*Yes*	46		36.2%
*No*	81		63,8%
			
***Caregivers' education***			
*Less than high school*	26		20%
*High School or higher*	104		80%
			
***HbA1c***			
*Adequate*	37		28.7%
*Inadequate*	92		71.3%
			
			
***Main Caregiver***			
*Mother*	101		77.7%
*Father*	21		16.2%
*Other*	8		6.2%
			
***BMI/age classification***			
*Eutrophy*	76		58.4%
*Risk of overweight*	3		2.3%
*Overweight*	37		28.5%
*Obesity*	11		8.5%
*Severe Obesity*	3		2.3%
			
***Height/Age classification***			
*Severely stunted*	4		3.1%
*Adequate*	126		96.9%
			
***EBIA Classification***			
*Food security*	42		32.6%
*Mild Food Insecurity*	65		50.4%
*Moderate food insecurity*	15		11.6%
*Severe food insecurity*	7		5.4%
			
***Overweight or Obesity or Severe Obesity***			
*Yes*	51		39.2%
*No*	79		60.8%

Missing data: Benefit receipt: n = 3; HbA1c: n = 1; EBIA classification: n = 1.

Regarding glycemic control, most patients had inadequate glycemic control (71.3%, n = 92), and the mean HbA1c was 8.15 ± 1.33%. According to anthropometric data, the majority were eutrophic (58.4%, n = 76) and had adequate height for their age (96.9%, n = 126). However, the frequency of overweight was 39.2% (n = 51).

The majority of the families interviewed (67.4%, n = 87) were in a food insecurity scenario. The predominant degree of FI was mild food insecurity (50.4%, n = 65), 11.6% (n = 15) were in moderate FI, and 5.4% (n = 7) were in severe FI.

When comparing sociodemographic factors with the EBIA classification, a statistically significant association (p-value < 0.05) was found between Food Insecurity and income, caregiver education, and receipt of benefits, as can be seen below in Table 2.

**Table 2:** Sociodemographic and clinical factors according to the EBIA classification.

Variables	Total n (%)		Food Security (n = 42; 32.6%)		Food Insecurity (n = 87; 67.4%)		p value
				
**Sex**							
Female	78 (60.5%)		28 (35.9%)		50 (64.1%)		0.343
Male	51 (39.5%)		14 (27.5%)		37 (72.5%)		
				
**Benefit Receipt**							
Yes	45 (35.7%)		7 (15.6%)		38 (84.4%)		0.030
No	81 (64.3%)		34 (42%)		47 (58%)		
				
**Caregivers' education**							
Less than high school	25 (19.4%)		3 (12%)		22 (88%)		0.017
High School or higher	104 (80.6%)		39 (37.5%)		65 (62.5%)		
				
**Income**							
< 1 minimum wage	34 (26.6%)		1 (2.9%)		33 (97.1%)		
1 - 3 minimum wages	60 (46.9%)		18 (30%)		42 (70%)		<0.001
3 - 5 minimum wages	29 (22.6%)		18 (62.1%)		11 (37.9%)		
> 5 minimum wages	5 (3.9%)		5 (100%)		0 (0%)		
				
**HbA1c**							
Adequate	37 (28,9%)		9 (24.3%)		28 (75.7%)		0.218
Inadequate	91 (71.1%)		33 (36.3%)		58 (63.7%)		
				
**Overweight or Obesity or Severe Obesity**							
Yes	50 (39.1%)		16 (32%)		34 (68%)		0.875
No	79 (60.9%)		26 (32.9%)		53 (67.1%)		

Missing data: Receipt of benefit: n = 4; Income: n = 2; HbA1c: n = 2; Overweight: n = 1; Sex: n = 1; Education: n = 1; EBIA classification: n = 1.

*At the time of the study, the Brazilian minimum wage was approximately USD 283,00/month.

## Discussion

According to the results found in this study, the prevalence of FI for families of children and adolescents with DM1 treated at the IPPMG outpatient clinic (67.4%, n = 87) was more than double that found in the last estimate made by IBGE for the Brazilian population, which was 27.6% of private households with some degree of FI, among the 78.3 million private households estimated in Brazil [18]

The prevalence of FI found in this study is also higher when compared to data from the 2019 National Study of Child Food and Nutrition (ENANI 2019), which found that 42.1% of the households studied had some degree of FI. Therefore, this high prevalence reinforces the need to develop food and nutrition actions and policies that take into account the most vulnerable populations, seeking to establish equity [19]

Consistent with this FI rate in the general population, studies show a high prevalence of FI among families of children with T1DM. Children living in this scenario have higher hospitalization rates and worse glycemic control. It is essential to better understand the factors that interfere with the food security of this population in order to consider actions that contribute to reducing FI [20,21]

Furthermore, a study of North American adults with diabetes showed that the combination of food insecurity and a poor-quality diet is related to worse HbA1c levels, regardless of other sociodemographic characteristics, adherence to other health care, and BMI, when compared to individuals with diabetes living in food security and with a high-quality diet. Therefore, it is clear that for good treatment, in addition to outlining strategies to improve other diabetes-related care, looking at the diet of these people is essential [4]

This study reinforces the findings in the literature that associate a lower level of education, receipt of benefits, and lower income as factors for Food Insecurity [22,23,24,25] Income is one of the main determinants of FI, since families with lower incomes have greater difficulty in acquiring and preparing nutrient-rich foods, causing challenges due to the lack of access to food and due to high prices [26]

In the study by Varela et al. (2016)[22], with Latino children up to 3 years of age living in the United States, low income and a low level of education were associated with Food Insecurity, with income being considered the main contributor to this condition. This review also cites the lack of nutritional education and training in food resource management as factors for FI.

Furthermore, in a study with adult patients with diabetes, it was shown that living below the poverty line was 5 times more prevalent among individuals living in FI than among those living in food security. With regard to education, the prevalence value was 2.5 times higher when comparing the same groups [4]

Regarding social benefits, Bueno et al. (2021) found greater Food Insecurity in families of children and adolescents aged 5 to 16 who received government assistance from the Bolsa Família Program (PBF) than in families that did not receive this benefit. This shows that this assistance is serving those who are, in fact, more vulnerable. Therefore, like other government programs such as the Food Acquisition Program (PAA) and the National School Feeding Program (PNAE), the PBF has proven to be important in reducing Food Insecurity, given that this benefit reduces poverty and improves Food and Nutrition Security conditions [24,27]

Although these programs have a high potential to impact the food insecurity ​scenario positively, the data suggest that they are still not enough to eliminate the problem completely. Therefore, it is necessary to expand and review income transfer programs and food subsidies to combat FI [28] Furthermore, Ronli et al. (2023)[29] addressed the need to create policies and programs that support food security, healthy eating, and quality health to avoid adverse outcomes in patients with diabetes.

Benefits recipient, caregiver education and income are related indicators of socioeconomic status; they represent distinct dimensions of social vulnerability. Income reflects household economic resources, caregiver education reflects human capital and health literacy, and receipt of social benefits reflects eligibility for governmental social protection programs.

For this reason, all variables were retained to provide a broader characterization of socioeconomic conditions.

Among the limitations of the study, despite serving a broad and heterogeneous population, the study was conducted in a single center, which may limit the generalizability of the findings to other regions of Brazil. In addition, the cross-sectional nature of the study prevents the determination of causality between the factors studied and FI.

To our knowledge, few studies have evaluated food insecurity and its associated factors among children and adolescents with type 1 diabetes mellitus in middle-income countries. This study contributes to the literature by demonstrating a high prevalence of food insecurity in a Brazilian pediatric T1DM population and identifying income, caregiver educational level, and receipt of social benefits as key factors associated with this condition. These findings reinforce the importance of integrating socioeconomic assessment into diabetes care and support the development of public policies aimed at reducing food insecurity among vulnerable families.

These results suggest the importance of planning intersectoral actions to create public policies and programs focused on reducing poverty, promoting food security, and adequate and healthy nutrition, as well as monitoring and improving programs with these objectives that already exist.

Furthermore, it is essential that public policies for the management of T1DM are not limited to the provision of inputs, but also include a strong focus on ensuring the right to access adequate and healthy food, given the impact on the management and prognosis of the disease. Even in a context of technological advances in the treatment of diabetes, the prevalence of food insecurity among families is still a significant challenge and requires urgent attention to promote equity in access to food and health. A greater focus on this issue is needed to achieve positive progress in this scenario.

## Funding sources

FAPERJ- 10.13039/501100004586Fundação Carlos Chagas Filho de Amparo à Pesquisa do Estado do Rio de Janeiro, Processo 298725.

## Data availability

The data that support the findings of this study are available from the corresponding author.

## Conflicts of interest

The authors declare no conflicts of interest.

## References

[1] American Diabetes Association Professional Practice Committee (2025). 2. Diagnosis and classification of diabetes: standards of care in diabetes-2025. Diabetes Care.

[2] Campos TF, Ramos S, Campos LF, Guimarães DB, Baptista DR, Gomes DL, et al. Terapia nutricional no diabetes tipo 1. Diretriz Oficial da Sociedade Brasileira de Diabetes. 2024. doi:10.29327/5412848.2024-4.

[3] Rodacki M, Teles M, Gabbay M, Montenegro R, Bertoluci M, Lamounier R. Classificação do diabetes. Diretriz Oficial da Sociedade Brasileira de Diabetes. 2023. doi:10.29327/557753.2022-1.

[4] Casagrande S.S., Bullard K.M., Siegel K.R., Lawrence JM. (2022). Food insecurity, diet quality, and suboptimal diabetes management among US adults with diabetes. BMJ Open Diabetes Res Care.

[5] Morais D.C., Dutra L.V., Franceschini S.C.C., Priore SE. (2014). Insegurança alimentar e indicadores antropométricos, dietéticos e sociais em estudos brasileiros: uma revisão sistemática. Cien Saude Colet.

[6] FAO, IFAD, UNICEF, WFP, WHO (2024).

[7] United Nations Children's Fund (UNICEF) (2024). https://www.unicef.org/media/157661/file/Child-food-poverty-2024.pdf.

[8] FAO, IFAD, UNICEF, WFP, WHO (2022).

[9] Instituto Brasileiro de Geografia e Estatística (2020). Pesquisa de Orçamentos Familiares 2017-2018: avaliação nutricional da disponibilidade domiciliar de alimentos no Brasil. Rio Jan.

[10] Medeiros A.R., Lima R.L., Medeiros L.B., Trajano F.P., Salerno A.A., Moraes R.M. (2017). Moderate and severe household food insecurity in families of people living with HIV/Aids: scale validation and associated factors. Cien Saude Colet.

[11] Weiser S.D., Palar K., Hatcher A.M., Young S.L., Frongillo E.A., Laraia BA. (2015). Food insecurity and public health.

[12] Seligman H.K., Berkowitz SA. (2019). Aligning programs and policies to support food security and public health goals in the United States. Annu Rev Public Health.

[13] Instituto Brasileiro de Geografia e Estatística (IBGE) (2024). https://biblioteca.ibge.gov.br/visualizacao/livros/liv102084.pdf.

[14] Segall-Corrêa A.M., Marin-Leon L., Melgar-Quiñonez H., Pérez-Escamilla R. (2014). Refinement of the Brazilian household food Insecurity measurement Scale: recommendation for a 14-item EBIA. Rev Nutr.

[15] Lohman T.G., Roche A.F., Martorell R. (1988).

[16] (2006). WHO Multicentre growth reference study group. WHO child growth standards based on length/height, weight and age. Acta Paediatr Suppl.

[17] World Health Organization (1995). Physical status: the use and interpretation of anthropometry. WHO Tech Rep Ser.

[18] Instituto Brasileiro de Geografia e Estatística (2024). https://biblioteca.ibge.gov.br/visualizacao/livros/liv102084.pdf.

[19] Universidade Federal do Rio de Janeiro (2021). https://enani.nutricao.ufrj.br/index.php/relatorios/.

[20] Marjerrison S., Cummings E.A., Glanville N.T., Kirk S.F., Ledwell M. (2011). Prevalence and associations of food insecurity in children with diabetes mellitus. J Pediatr.

[21] Mendoza J.A., Haaland W., D'Agostino R.B., Martini L., Pihoker C., Frongillo E.A. (2018). Food insecurity is associated with high-risk glycemic control and higher health care utilization among youth and young adults with type 1 diabetes. Diabetes Res Clin Pr.

[22] Varela E.G., McVay M.A., Shelnutt K.P., Mobley AR. (2023). The determinants of food insecurity among Hispanic/Latinx households with young children: a narrative review. Adv Nutr.

[23] Santos I.N., Damião J.J., Fonseca M.J., Cople-Rodrigues C.S., Aguiar OB. (2019). Food insecurity and social support in families of children with sickle-cell disease. J Pediatr (Rio J).

[24] Bueno M.C., Franco J.G., Leal G.V.S., VR Kirsten (2021). [Food insecurity and the relationship with social, economic and nutritional factors in rural school students]. Cad Saude Colet.

[25] Jesus JG, Hoffmann R, Miranda SHG. Food insecurity, poverty and income distribution in Brazil. 2024;62(4):e281936.

[26] Eicher-Miller H.A., Graves L., McGowan B., Mayfield B.J., Connolly B.A., Stevens W. (2023). A scoping review of household factors contributing to dietary quality and food security in low-income households with school-age children in the United States. Adv Nutr.

[27] Kopruszynski C.P., Costa VMH. (2016). Programas de transferência condicionada de renda e segurança alimentar e nutricional. Segur Aliment Nutr.

[28] Lignani J.B., Palmeira P.A., Antunes M.M.L. (2020). Salles-Costa R. Relationship between social indicators and food insecurity: a systematic review. Rev Bras Epidemiol.

[29] Levi R., Bleich S.N., Seligman HK. (2023). Food insecurity and diabetes: overview of intersections and potential dual solutions. Diabetes Care.

